# A selective inhibitor of the NLRP3 inflammasome as a potential therapeutic approach for neuroprotection in a transgenic mouse model of Huntington’s disease

**DOI:** 10.1186/s12974-022-02419-9

**Published:** 2022-02-26

**Authors:** Kai-Po Chen, Kuo-Feng Hua, Fu-Ting Tsai, Ting-Yu Lin, Chih-Yuan Cheng, Ding-I. Yang, Hsien-Ta Hsu, Tz-Chuen Ju

**Affiliations:** 1 Department of Animal Science and Biotechnology, Tunghai University, Taichung, Taiwan; 2 Department of Biotechnology and Animal Science, National Ilan University, Ilan, Taiwan; 3 Department of Medical Research, China Medical University Hospital, China Medical University, Taichung, Taiwan; 4 Institute of Brain Science and Brain Research Center, National Yang Ming Chiao Tung University, Taipei, Taiwan; 5 Division of Neurosurgery, Taipei Tzu Chi Hospital, Buddhist Tzu Chi Medical Foundation, New Taipei City, Taiwan; 6 School of Medicine, Buddhist Tzu Chi University, Hualien, Taiwan; 7 National Defense Medical Center, Taipei, 114201 Taiwan; 8 Department of Animal Science and Biotechnology, Tunghai University, No. 1727, Sec. 4, Taiwan Blvd., Xitun Dist., Taichung City, 40704 Taiwan

**Keywords:** Huntington’s disease (HD), Interleukin‐1β (IL‐1β), Mutated huntingtin (mHTT), Nucleotide oligomerization domain-like receptor protein 3 inflammasome (NLRP3 inflammasome)

## Abstract

**Background:**

Huntington’s disease (HD) is a neurodegenerative disorder caused by the expansion of the CAG repeat in the *huntingtin* (*HTT*) gene. When the number of CAG repeats exceeds 36, the translated expanded polyglutamine-containing HTT protein (mutant HTT [mHTT]) interferes with the normal functions of many cellular proteins and subsequently jeopardizes important cellular machineries in major types of brain cells, including neurons, astrocytes, and microglia. The NACHT, LRR, and PYD domain-containing protein 3 (NLRP3) inflammasome, which comprises NLRP3, ASC, and caspase-1, is involved in the activation of IL-1β and IL-18 and has been implicated in various biological functions. Although the existence of the NLRP3 inflammasome in the brain has been documented, the roles of the NLRP3 inflammasome in HD remain largely uncharacterized. MCC950 is a highly selective and potent small-molecule inhibitor of NLRP3 that has been used for the treatment of several diseases such as Alzheimer’s disease. However, whether MCC950 is also beneficial in HD remains unknown. Therefore, we hypothesized that MCC950 exerts beneficial effects in a transgenic mouse model of HD.

**Methods:**

To evaluate the effects of MCC950 in HD, we used the R6/2 (B6CBA-Tg[HDexon1]62Gpb/1J) transgenic mouse model of HD, which expresses exon 1 of the human *HTT* gene carrying 120 ± 5 CAG repeats. Male transgenic R6/2 mice were treated daily with MCC950 (10 mg/kg of body weight; oral administration) or water for 5 weeks from the age of 7 weeks. We examined neuronal density, neuroinflammation, and mHTT aggregation in the striatum of R6/2 mice vs. their wild-type littermates. We also evaluated the motor function, body weight, and lifespan of R6/2 mice.

**Results:**

Systematic administration of MCC950 to R6/2 mice suppressed the NLRP3 inflammasome, decreased IL-1β and reactive oxygen species production, and reduced neuronal toxicity, as assessed based on increased neuronal density and upregulation of the NeuN and PSD-95 proteins. Most importantly, oral administration of MCC950 increased neuronal survival, reduced neuroinflammation, extended lifespan, and improved motor dysfunction in R6/2 mice.

**Conclusions:**

Collectively, our findings indicate that MCC950 exerts beneficial effects in a transgenic mouse model of HD and has therapeutic potential for treatment of this devastating neurodegenerative disease.

**Supplementary Information:**

The online version contains supplementary material available at 10.1186/s12974-022-02419-9.

## Background

Huntington’s disease (HD) is a neurodegenerative disease that is inherited in an autosomal dominant manner and mainly affects neurons in the brain, particularly in the striatum. HD also affects the cortex, which controls thinking, comprehension, and memory. Therefore, HD is often accompanied by progressive cognitive, motor, and mental impairment [1, 2]. The cause of HD is the abnormal expansion of the CAG repeat located in the first exon of the *huntingtin* (*HTT*) gene on chromosome 4, resulting in an abnormal gene product (mutated HTT [mHTT]) [3–5]. mHTT accumulates abnormally in neurons and neuroglial cells [3, 6–8]. Therefore, the cellular physiological characteristics of HD include excitotoxicity, dysregulated energy metabolism, transcriptional changes, mitochondrial dysfunction, impaired autophagy, axonal degeneration, oxidative stress, and increased inflammation [2, 6, 9–11].

The NACHT, LRR, and PYD domain-containing protein 3 (NLRP3) inflammasome is an intracellular protein complex that activates caspases. Currently, NLRP3 is one of the most studied NLR family members. The NLRP3 inflammasome consists of three components. The first component is NLRP3, which mainly controls inflammasome specificity and activity. The NLRP protein contains leucine-rich repeats and functions similarly to Toll-like receptors. The second component is the scaffold protein ASC. NLRP3 binds to ASC, and ASC binds to the third component of the inflammasome, pro-caspase-1 [12]. These three components combine to form the NLRP3 inflammasome. In turn, activation of the NLRP3 inflammasome leads to the cleavage of pro-caspase-1 into caspase-1, before the cleavage of pro-interleukin-1β (pro-IL-1β) and pro-IL-18 into interleukin-1β (IL-1β) and IL-18, respectively [13]. Therefore, IL-1β and IL-18 are the final products of the activity of the NLRP3 inflammasome. NLRP3 inflammasome activation requires two signals, namely the priming signal and the activation signal. The priming signal mainly stems from the Toll-like receptor and is responsible for producing the proteins that are required for NLRP3 inflammasome activation, such as NLRP3 and pro-IL-1β. Previous studies found that the activation signal may be caused by metabolic abnormalities or protein aggregation, such as hyperglycemia, hyperlipidemia, hypercholesterolemia, and hyperuricemia [14–16]. More importantly, activation of the NLRP3 inflammasome is also associated with neurodegenerative diseases, such as Alzheimer’s disease (AD) and Parkinson’s disease (PD). Many recent studies found that key factors that cause neurodegenerative diseases, such as 1-methyl-4-phenyl-1,2,3,6-tetrahydropyridine (MPTP), amyloid-β (Aβ), and Tau, are also activation signals for the NLRP3 inflammasome [17–20]. Regulation of the NLRP3 inflammasome can alleviate the disease course to varying degrees, and even block key mechanisms in certain diseases. This shows that the NLRP3 inflammasome may be an important marker in the treatment of neurodegenerative diseases.

MCC950 is a highly selective and potent small-molecule inhibitor of NLRP3 that was first applied to attenuate experimental autoimmune encephalomyelitis [21]. However, MCC950 is not currently used in the treatment of HD. The mean life expectancy of patients with HD after diagnosis is 15–18 years, and the disease course cannot be stopped or reversed after its onset. At present, there is no treatment for HD. In 2008, the U.S. Food and Drug Administration (FDA) approved Nitoman® (generic name: tetrabenazine; Apotex Inc., Headquarters Toronto, Ontario, Canada) for the treatment of involuntary movements in HD. This medication was the first drug to be approved for the treatment of this disease and can effectively treat abnormal limb movements. Two pharmaceutical antisense oligonucleotides (ASOs) have been used in HD research, and dose-dependent reductions in the concentrations of mHTT have been reported [22]. However, phase III clinical trials on ASOs have been halted in relation to gene-targeting therapies for HD. In addition to tetrabenazine and ASOs, other drugs have been suggested for use in HD; however, the existing drugs can only alleviate, rather than stop, neuro degeneration. The main mechanisms of action of these drugs involve the inhibition of monoamines such as serotonin, dopamine, and norepinephrine, release from nerve terminals, and the inhibition of vesicular monoamine transporter 2, which subsequently reduces uptake of monoamines into synaptic vesicles along with depletion of monoamine storage.

As the mechanisms of neurodegeneration in HD are still not completely clear, it is extremely important to further examine the pathogenesis of HD. An excessive inflammatory reaction is one of the important factors that cause cell death in HD. The NLRP3 inflammasome plays an important role in inflammatory reactions and is involved in the pathogenesis of several neurodegenerative diseases such as AD and PD. However, the direct role of the NLRP3 inflammasome in HD has not been explored. Therefore, the NLRP3 inflammasome inhibitor MCC950 was selected in this study to determine the role of the NLRP3 inflammasome in HD pathogenesis. The results of this study will aid future basic medical studies of neurodegenerative diseases and the R&D of clinical drugs.

## Materials and methods

### Cell culture

Striatal progenitor cell lines (STHdh^Q7^ and STHdh^Q109^) were generous gifts from Dr. Elena Cattaneo (Department of Pharmacological Sciences and Centre for Stem Cell Research, University of Milano, Italy) and Yijuang Chern (Institute of Biomedical Sciences, Academia Sinica, Nankang, Taipei, Taiwan). The conditionally immortalized striatal neuronal progenitor cells STHdh^Q7^, which express endogenous normal HTT comprising seven glutamine residues and are referred to as wild-type (WT) striatal cells, and STHdh^Q109^ cells, which are derived from homozygous STHdh^Q109^ knock-in mice expressing mHTT containing 109 glutamine residues and are referred to as mutant striatal cells, were maintained in an incubation chamber at 33 °C and 5% CO_2_ in Dulbecco’s modified Eagle’s medium (DMEM; Thermo Fisher Scientific Inc., Waltham, MA, USA) supplemented with 10% fetal bovine serum (FBS; Thermo Fisher Scientific Inc., Waltham, MA, USA) [23, 24]. Cells with a passage number < 20 were exclusively used in the present study. BV2 cells (mouse, C57BL/6; brain, microglial cells) were purchased from the American Type Culture Collection (Rockville, MD). Cells were propagated in DMEM supplemented with 10% FBS at 37 °C in a 5% CO_2_ incubator.

### Cell death assays

The death of STHdh^Q7^ and STHdh^Q109^ cells was quantified using the Cell Counting Kit-8 (CCK-8; Thermo Fisher Scientific Inc., Waltham, MA, USA). For the CCK-8 assay, a volume of CCK-8 reagent corresponding to 10% of the volume of the medium in the well was added, followed by incubation at 33 °C in a 5% CO_2_ incubator for 1 h. The absorbance was measured at 450 nm using a microplate reader (OPTImax tunable plate reader; Molecular Devices, Wokingham, UK).

### Sodium dodecyl sulfate polyacrylamide gel electrophoresis and Western blotting

Western blot analysis was performed as described previously [11]. Briefly, cellular proteins were extracted in TNE buffer (50 mM Tris–HCl [pH 7.4], 100 mM NaCl, 0.1 mM EDTA, and 1% Triton X-100). Proteins were separated by electrophoresis in 10–15% polyacrylamide gels and transferred onto polyvinylidene difluoride membranes (Millipore, Billerica, MA, USA). The dilutions of the primary antibodies used in the present study were as follows: anti-NLRP3, anti-ASC, anti-caspase-1, anti-PSD-95, and anti-NeuN antibodies at 1:1000 (all from Cell Signaling Technology, Danvers, MA, USA); anti-glial fibrillary acidic protein (1:1000, GFAP), anti-ionized calcium-binding adaptor molecule-1 (1:500, Iba-1), and anti-mHTT antibodies (1:500 all from Millipore, Billerica, MA, USA). The immunoreactive signals on the blots were detected using an enhanced chemiluminescence detection system (PerkinElmer Life and Analytical Sciences, Boston, MA, USA).

### Animals and treatment

Male transgenic R6/2 mice (B6CBA-Tg[HDexon1]62Gpb/1J) expressing exon 1 of the human *HTT* gene carrying 120 ± 5 CAG repeats were obtained from the Jackson Laboratories (Bar Harbor, ME, USA). Posterity was identified using PCR genotyping and sequencing of genomic DNA using primers located in the transgene (5′-CCGCTCAGGTTCTGCTTTTA-3′ and 5′-GGCTGAGGAAGCTGAGGAG-3′). All animal experiments were approved by the Tunghai University Animal Ethics Committee (approval number 108–39). Animals were reared at the Tunghai University Animal Care Facility under a 12 h light/dark cycle. The ambient temperature was maintained at 25 °C ± 2 °C and the animals had ad libitum access to food and water. All animal experiments were performed according to good laboratory practice. Experiments were planned and performed according to the 3Rs principle, which comprise the reduction of animal suffering and number of mice used. Each group of 16–20 mice received daily treatment with water (control group) or MCC950 (10 mg/kg of body weight, oral administration) for 5 weeks from the age of 7 weeks. Mice were weighed every week. Behavioral assays were performed between 5 and 12 weeks of age. After the experiment, the weight-loss trends and survival rate of mice were analyzed. Animals were killed and tissues were collected for subsequent analyses.

### Behavioral tests

#### Rotarod performance

Motor coordination was assessed using a rotarod apparatus (UGO BASILE, Comerio, Italy) at a constant speed [25, 26]. All mice were tested three times per week. Each test session comprised three trials for each mouse. The latency to fall from the rotating rod, up to a maximum of 2 min, was recorded for each trial. The weekly maximum performance for each mouse was used for statistical analysis [11].

#### Clasping

At 12 weeks of age, the positioning of the limb-clasping response was tested. Briefly, mice were suspended by their tails from a height of 50 cm for 30 s [27, 28]. A limb-clasping response was defined as the withdrawal of any limb to the torso for > 2 s. A score of 0 was assigned if the hind limbs were consistently splayed outward, away from the abdomen. A score of 1 was assigned if one hind limb was retracted toward the abdomen for more than 50% of the 10-s observation period. A score of 2 was assigned if both hind limbs were partially retracted toward the abdomen for more than 50% of the 10-s observation period. Finally, a score of 3 was assigned if the hind limbs were entirely retracted and touching the abdomen for more than 50% of the 10-s observation period [29].

### Enzyme-linked immunosorbent assay

BV2 microglia cells were treated with lipopolysaccharide (LPS; 1 µg/mL) for 4 h, after which they were incubated with or without MCC950 for 2 h before stimulation with ATP (1 mM) for 24 h. Levels of IL-1β, IL-18, and TNF in the culture medium were measured using an enzyme-linked immunosorbent assay (ELISA) according to the manufacturer’s protocol. Briefly, the 96-well microplates were coated overnight with anti-IL-1β, anti-IL-18, or anti-TNF antibody then blocked with 1% bovine serum albumin (BSA). Standards or culture medium (100 µL) were added to the microplates and incubated at room temperature for 2 h; this was followed first by incubation for 2 h with the biotin-conjugated detection antibody then by incubation for 30 min with 100 µL of streptavidin–horseradish peroxidase plus substrate for signal development. Finally, 100 µL of stop solution was added to each well and the OD_450_ was measured using an ELISA microplate reader (Bio-Tek Instruments, Winooski, VT, USA) [30].

### Measurement of glutathione levels

The cells were washed twice in phosphate-buffered saline and lysed in 15 mM Tris, pH 7.4 before the intracellular levels of glutathione were determined using a fluorimetric assay. Glutathione levels were measured in samples after the addition of *ortho*-phthalaldehyde (1 mg/mL of methanol) and 100 mM NaH_2_PO_4_. After a 15-min incubation, the fluorescence was measured using an excitation wavelength of 350 nm and an emission wavelength of 420 nm. The results were calculated as relative fluorescence units per mg of protein and are expressed as a percentage of the WT cells or control.

### Immunohistochemistry and quantitation

Brain sections were cut and stored as described above for the reactive oxygen species (ROS) measurements followed by immunohistochemical staining as described previously [31]. Briefly, brain sections were incubated overnight with the appropriate primary antibody in phosphate-buffered saline containing 5% normal goat serum at 4 °C; this was followed by incubation with the corresponding secondary antibody for 2 h at room temperature. The following primary antibodies and concentrations were used: anti-NLRP3 (Adipogen International, San Diego, CA, USA), anti-NeuN (Cell Signaling Technology, Danvers, MA, USA), anti-GFAP, anti-Iba I, and anti-EM48 (all from Millipore, Billerica, MA, USA). The secondary antibodies were conjugated to Alexa Fluor 488, Alexa Fluor 568, or Alexa Fluor 633 (Thermo Fisher Scientific Inc., Waltham, MA, USA). Nuclei were stained with 4′,6-diamidino-2-phenylindole (DAPI). Slides were mounted using Vectashield (Vector Laboratories, Burlingame, CA, USA). To determine the number of neurons in the striatum, nine frames from three sections spaced evenly throughout the striatum (interaural 5.34 mm/bregma 1.54 mm to interaural 3.7 mm/bregma − 0.1 mm) were analyzed for each animal by an investigator blinded to the experimental conditions; at least 500 cells from each animal were counted and measured. For quantitation of mHTT, images were acquired using laser confocal microscopy (LSM810; Carl Zeiss MicroImaging, Thornwood, NY, USA) and analyzed using the MetaMorph imaging system (Universal Imaging, Westchester, PA, USA)*.*

### Statistical analysis

The results are expressed as the mean ± standard error of the mean (SEM) of triplicate measurements. Each experiment was repeated at least three times to confirm the reproducibility of the findings. Comparisons among multiple groups were analyzed by one-way analysis of variance followed by Dunnett’s post hoc test. Differences between treatment means were considered statistically significant at *P* < 0.05.

## Results

### MCC950 significantly reduces cytotoxicity in striatal progenitor and BV2 cells

**Fig. 1:**
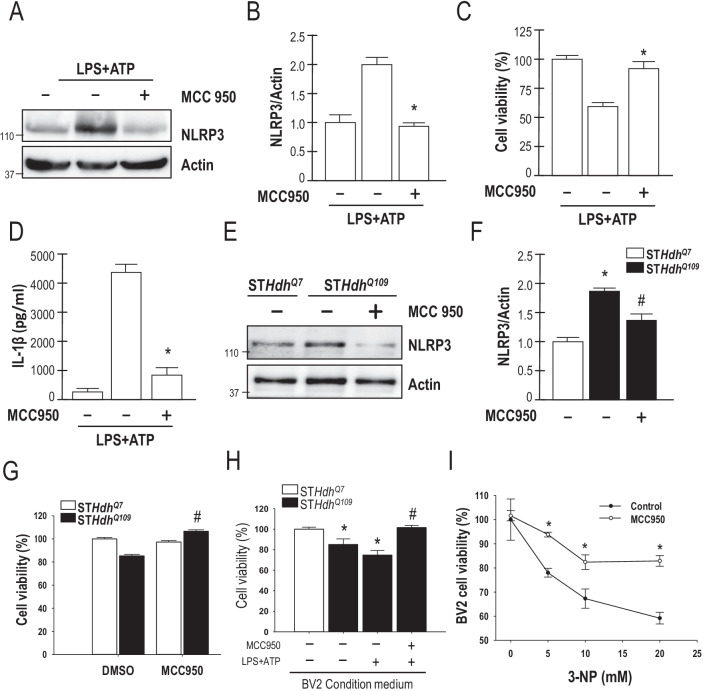
MCC950 markedly reduces cytotoxicity in striatal progenitor cells and BV2 microglial cells. **A**, **B** BV2 microglia were incubated for 4 h with LPS (1 μg/mL) followed by incubation with MCC950 (1 μM) for 2 h. The cells were then incubated with ATP (1 mM, 24 h). Total lysates of BV2 microglial cells were assessed by Western blot analysis to determine the levels of the NLRP3 and actin proteins. The molecular mass is indicated in kilodaltons. **C**, **D** BV2 microglia were incubated for 4 h with LPS (1 μg/mL) followed by incubation with MCC950 (1 μM) for 2 h. The cells were then incubated with ATP (1 mM for 24 h). Cell survival (**C**) and IL-1β expression levels (**D**) were measured using the CCK-8 assay and ELISA, respectively. The values of the indicated cells were normalized to those of untreated BV2 cells. *P < 0.05 compared to LPS/ATP treated cells (*n* = 3). **E**, **F** ST*Hdh*^Q109^ cells were incubated for 24 h with MCC950 (1 μM). Total lysates of ST*Hdh*^*Q7*^ and ST*Hdh*^*Q109*^ cells were assessed using Western blot analysis. **G** ST*Hdh*^*Q7*^ and ST*Hdh*^Q109^ cells were incubated for 24 h with MCC950 (1 μM). Cell death was quantified using the CCK-8 assay; the values of the indicated cells were normalized to those of untreated ST*Hdh*^*Q7*^ cells. The data are presented as the mean ± SEM from three independent experiments. *P < 0.05, ST*Hdh*^*Q7*^ vs. ST*Hdh*^*Q109*^ cells; ^#^*P* < 0.05 vs. untreated ST*Hdh*^*Q109*^ cells. **H** BV2 cells were incubated with LPS (1 µg/mL for 4 h) with or without MCC950 for 2 h before stimulation with ATP (1 mM for 24 h). The BV2 medium was then collected and used to culture the ST*Hdh*^Q109^ cells for an additional 24 h. ST*Hdh*^Q7^ and ST*Hdh*^*Q109*^ cell viability was determined by CCK-8 assay. The data are presented as the mean ± SEM from three independent experiments. *P < 0.05, ST*Hdh*^*Q7*^ vs. ST*Hdh*^*Q109*^ cells; ^#^*P* < 0.05 vs. untreated ST*Hdh*^*Q109*^ cells. **I** BV2 cells were treated with or without MCC950 (1 μM) and 3-NP (5, 10, and 20 mM) for 24 h. The cell viability was determined using the CCK-8 assay. Data are presented as the mean ± SEM from three independent experiments. *P < 0.05 compared with controls (*n* = 3)

Previous studies showed that MCC950 has extremely high specificity and is capable of effectively inhibiting NLRP3 and its downstream products [21]. Therefore, we used MCC950 to inhibit NLRP3 expression in HD models. Extracellular ligands (such as LPS and ATP) can activate the NLRP3 inflammasome [32, 33]. Thus, we treated mouse BV2 cells (mouse, C57BL/6; brain, microglial cells) that were LPS-primed by incubation with MCC950 before stimulation with a P2X7 activator (ATP) of the NLRP3 inflammasome. MCC950 was found to inhibit NLRP3 expression in BV2 cells (Fig. 1A, B). To determine whether NLRP3 activation was detrimental, we evaluated whether inhibition of NLRP3 by MCC950 affected BV2 cell viability using a CCK-8 assay. As shown in Fig. 1C and D, an ELISA indicated that inhibition of NLRP3 by MCC950 enhanced BV2 microglial cell viability and reduced IL-1β production. In addition, we used striatal progenitor cells to analyze the function of NLRP3 in HD. Expression of NLRP3 was greatly enhanced in ST*Hdh*^Q109^ cells compared with ST*Hdh*^Q7^ cells. We then investigated the influences of MCC950 on cell viability using the CCK-8 assay. ST*Hdh*^Q109^ cells were incubated with or without 0.1, 1, 2, or 5 μm MCC950 for 24 h (Additional file 1: Fig. S1). As predicted, MCC950 significantly reduced the NLRP3 levels in ST*Hdh*^Q109^ cells (Fig. 1E, F, and Additional file 1: Fig. S1). To determine whether NLRP3 plays a detrimental role in HD, we next evaluated whether inhibition of NLRP3 by MCC950 affected the viability of striatal progenitor cells. As shown in Fig. 1G, inhibition of NLRP3 by MCC950 increased the survival of ST*Hdh*^Q109^ cells. However, a high dose of MCC950 enhanced the death of ST*Hdh*^Q109^ cells (Fig. 1H). We then tested whether the activation of NLRP3 with the release of cytokines (IL-1β) by BV2 cells altered the viability of striatal progenitor cells. We collected the BV2 medium and cultured ST*Hdh*^Q7^ and ST*Hdh*^Q109^ cells in this medium. LPS-stimulated BV2 medium led to ST*Hdh*^Q109^ cell death, which was inhibited by MCC950 (Fig. 1H). Subsequently, we treated BV2 cells with 3-nitropropionic acid (3-NP), an irreversible inhibitor of mitochondrial complex II to induce HD in BV2 cells. As shown in Fig. 1I, MCC950 blocked the death of BV2 cells induced by 3-NP. Thus, MCC950 not only inhibited the NLRP3 inflammasome but also protected striatal cells from mHTT-mediated toxicity. Overall, these results highlight a relationship between mHTT and NLRP3 and demonstrate that inhibition of the NLRP3 inflammasome by MCC950 is beneficial to HD striatal progenitor cells in vitro*.*

### MCC950 inhibits NLRP3 inflammasome assembly in a transgenic mouse model (R6/2) of HD

**Fig. 2:**
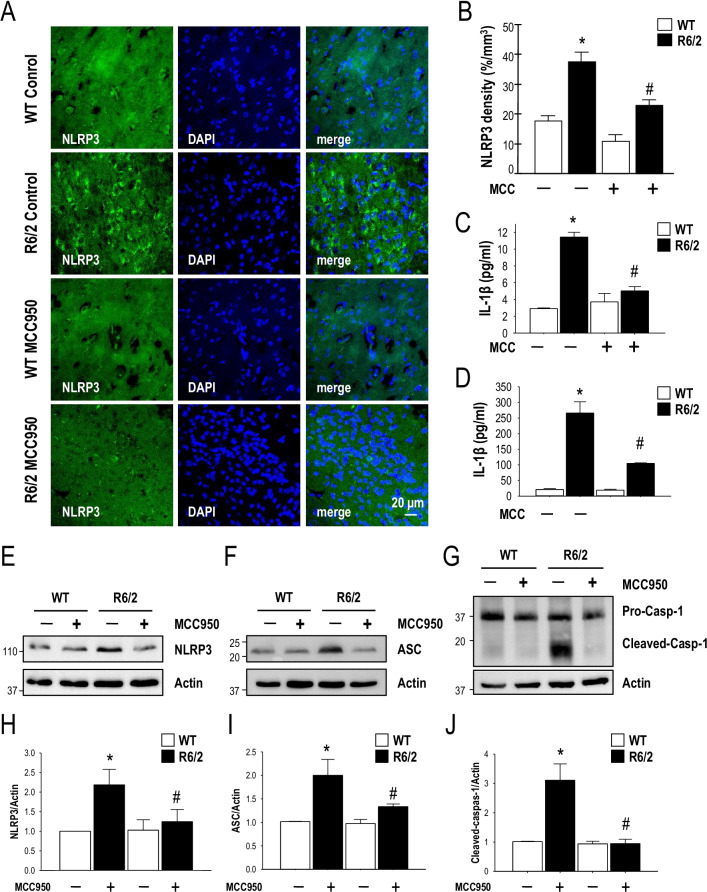
MCC950 triggers NLRP3 inflammasome assembly in a transgenic mouse model (R6/2) of HD. Mice were treated daily with MCC950 (10 mg/kg of body weight; oral administration) or water for 5 weeks from the age of 7 weeks. The number of NLRP3-positive cells (identified by the expression of NLRP3; green, **A**) in the indicated mice (water-treated WT mice [*n* = 6], water-treated R6/2 mice [*n* = 6], MCC950-treated WT mice [*n* = 6], and MCC950-treated R6/2 mice [*n* = 6]) was quantified. Nuclei were stained with DAPI (blue). The histograms show the number of NLRP3-positive cells in the striatum (**B**). At least 500 cells from each animal were counted. The levels of IL-1β in the serum (**C**) and striatum (**D**) were measured by ELISA (*n* = 3–6 for each condition). The data are presented as the mean ± SEM. *P < 0.05, between WT and R6/2 mice; ^#^*P* < 0.05 vs. water-treated R6/2 mice. Scale bars, 20 μm. **E**–**J** Striatal lysates were analyzed using Western blotting. The results were normalized to those of actin. Data are presented as the mean ± SEM from three independent experiments. The molecular mass is indicated in kilodaltons. *P < 0.05, between WT and R6/2 mice; ^#^*P* < 0.05 vs. water-treated R6/2 mice

To characterize the expression level of NLRP3 in HD, we first evaluated the expression of NLRP3 in the brains of HD mice using an immunofluorescence staining technique. We found that the expression level of NLRP3 was significantly increased in the striatum of R6/2 mice, but not in that of the WT mice. MCC950 is a small-molecule inhibitor of the NLRP3 inflammasome that can freely cross the blood–brain barrier. To evaluate whether NLRP3 is an important pathogenic factor in HD, R6/2 mice (7 weeks of age, *n* = 16–20 per group) were treated daily with MCC950 (10 mg/kg of body weight) or water via oral administration for 5 weeks from the age of 7 weeks. Consistent with the findings obtained for ST*Hdh*^Q109^ cells, oral administration of MCC950 suppressed the elevated NLRP3 levels in the striatum of R6/2 mice (Fig. 2A, B, E, H). Activation of the NLRP3 inflammasome results in the release of high levels of IL-1β and IL-18, thereby worsening neuroinflammation. Long-term neuroinflammation activates microglia to produce a considerable amount of IL-1β, resulting in neurotoxicity and accelerating neuronal death [34]. Therefore, we next assessed the IL‐1β level in the serum and striatum of HD mice. Oral administration of MCC950 blocked IL-1β secretion in the serum (Fig. 2C) and striatum of R6/2 mice (Fig. 2D). In addition, MCC950 markedly reduced IL-18 secretion in R6/2 mice (Additional file 1: Fig. S2). As shown in Fig. 2F and I, inhibition of NLRP3 by MCC950 significantly reduced the expression level of ASC in the striatum of R6/2 mice. It has been demonstrated that the NLRP3 inflammasome particles are released from caspase‐1-activated macrophages and act as a particulate danger signal that amplifies the inflammatory response [35]. Consistent with the importance of caspase‐1 in the activation of NLRP3, we found that MCC950 significantly reduced the activation of caspase‐1 in R6/2 mice (Fig. 2G, J). Taken together, these data suggest that MCC950 significantly inhibits the production of components of the NLRP3 inflammasome (i.e., NLRP3, ASC, and caspase‐1) in R6/2 mice.

### Blockage of the NLRP3 inflammasome delays disease progression in a transgenic mouse model (R6/2) of HD

**Fig. 3:**
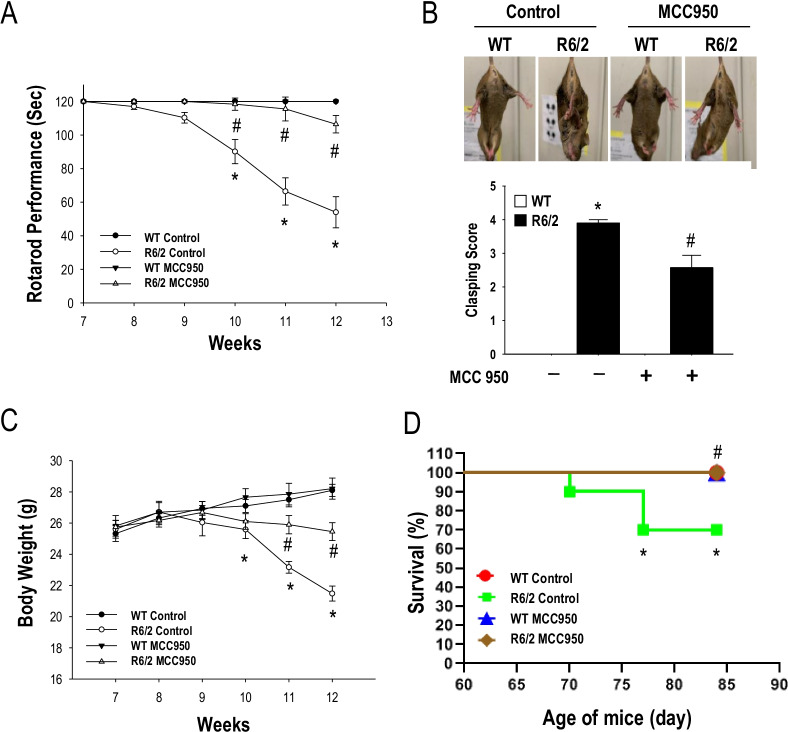
MCC950 markedly reduces disease progression in a transgenic mouse model (R6/2) of HD. Mice were treated daily with MCC950 (10 mg/kg of body weight; oral administration) or water for 5 weeks from the age of 7 weeks. Rotarod performance (**A**), clasping (**B**), and body weight (**C**) (*n* = 16–20 for each condition) were assessed. Data are presented as the mean ± SEM. *P < 0.05, WT vs. R6/2 mice; ^#^*P* < 0.05 vs. water-treated R6/2 mice. **D** Survival assessment (*n* = 16–20 for each condition; *P* < 0.05, Kaplan–Meier survival analysis)

To assess whether NLRP3 is an important pathogenic factor for HD, we examined the effect of long-term treatment with MCC950 on disease progression in a transgenic mouse model (R6/2) of HD. R6/2 mice received oral administration of MCC950 (10 mg/kg of body weight) or water for 5 weeks from the age of 7 weeks. Disease progression was assessed based on rotarod performance, body weight, clasping score, and lifespan. As shown in Fig. 3, oral administration of MCC950 markedly mitigated the motor dysfunction, as assessed by rotarod performance (Fig. 3A) and clasping score (Fig. 3B) in R6/2 mice. Most importantly, long-term treatment with MCC950 significantly increased the body weight (Fig. 3C) and extended the lifespan (Fig. 3D) of R6/2 mice. These findings support the hypothesis that elevated NLRP3 plays an important pathogenic role in a transgenic mouse model of HD.

### Blockage of the NLRP3 inflammasome in R6/2 mice reduces neuronal toxicity and mHTT aggregation

**Fig. 4:**
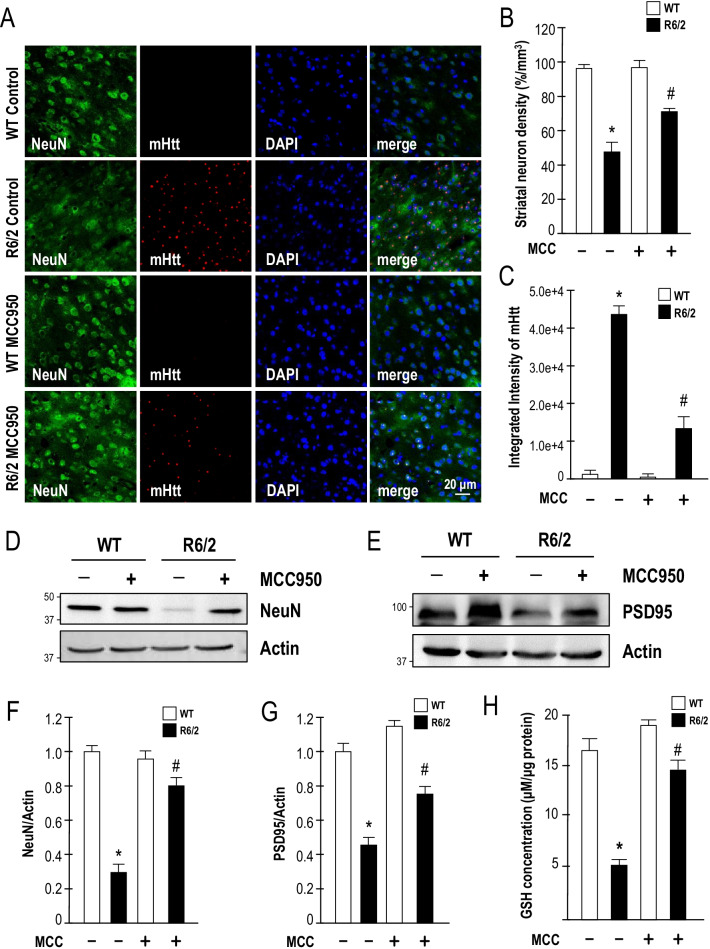
MCC950 significantly reduces neuronal loss and mHTT aggregation in a transgenic mouse model (R6/2) of HD. Mice were treated daily with MCC950 (10 mg/kg of body weight; oral administration) or water for 5 weeks from the age of 7 weeks. **A** Brain sections of 12-week-old mice were stained for NeuN and EM48. The number of neurons (as identified by the expression of NeuN; green) and the level of mHTT aggregation (EM48; red) in the striatum of the indicated mice (water-treated WT mice [*n* = 6], water-treated R6/2 mice [*n* = 6], MCC950-treated WT mice [*n* = 6], and MCC950-treated R6/2 mice [*n* = 6]) were quantified. Nuclei were stained with DAPI (blue). The histograms show the number of striatal neurons (**B**) and the integrated intensity of mHTT (**C**). At least 500 cells from each animal were counted. Data are presented as the mean ± SEM*.* Scale bars, 20 μm. *P < 0.05, between WT and R6/2 mice; ^#^*P* < 0.05 vs. water-treated R6/2 mice. **D**–**G** Striatal lysates were analyzed using Western blot analysis. The molecular mass is indicated in kilodaltons. Results were normalized to those of actin. *P < 0.05, between WT and R6/2 mice; ^#^*P* < 0.05 vs. water-treated R6/2 mice. **H** Striatal lysates were analyzed using a glutathione assay. *P < 0.05, between WT and R6/2 mice; ^#^*P* < 0.05 vs. water-treated R6/2 mice

Consistent with the improved lifespan and motor dysfunction, the number of neurons in the striatum was also markedly increased in the striatum of MCC950-treated R6/2 mice (Fig. 4A, B). Immunofluorescence staining showed that long-term treatment with MCC950 significantly reduced the number of mHTT aggregates in the striatum of HD mice (Fig. 4A, C). As shown in Fig. 4D–G, inhibition of NLRP3 by MCC950 significantly enhanced the expression levels of NeuN and PSD-95 in R6/2 mice. Taken together, these data suggest that NLRP3 plays a critical role in a transgenic mouse model of HD. Previous studies found that mitochondrial ROS synthesis is involved in the NLRP3 inflammasome activation pathways [12, 36, 37]. Therefore, we performed experiments to detect ROS production in striatal cells and R6/2 mice. Our findings were consistent with those of several previous studies that showed more severe oxidative damage in the brains of HD mice [38, 39]; daily treatment with the NLRP3 inhibitor MCC950 for 5 weeks from the age of 7 weeks markedly enhanced the level of glutathione in the striatum of R6/2 mice (Fig. 4H). Taken together, our data suggest that MCC950 protects striatal neurons against mHTT-mediated toxicity by interfering with the detrimental action of the NLRP3 inflammasome activation pathways in a transgenic mouse model of HD.

### Inhibition of NLRP3 reduces microglial and astrocytic activation in a transgenic mouse model (R6/2) of HD

**Fig. 5:**
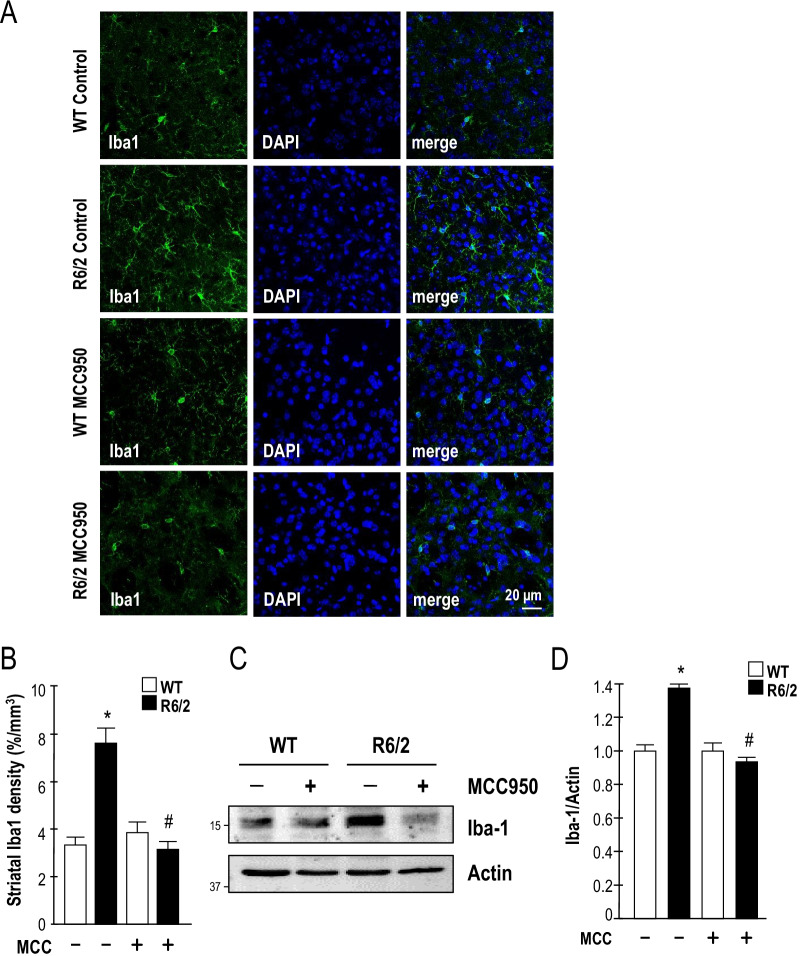
MCC950 inhibits microglial activation in a transgenic mouse model (R6/2) of HD. Mice were treated daily with MCC950 (10 mg/kg body weight; oral administration) or water for 5 weeks from the age of 7 weeks. **A** Brain sections of 12-week-old mice were stained against Iba-1. The number of microglia (identified by the expression of Iba-1; green) in the striatum of the indicated mice (water-treated WT mice [*n* = 6], water-treated R6/2 mice [*n* = 6], MCC950-treated WT mice [*n* = 6], and MCC950-treated R6/2 mice [*n* = 6]) were quantified. Nuclei were stained with DAPI (blue). The histograms show the integrated intensity of striatal microglia (**B**). At least 500 cells from each animal were counted. Data are presented as the mean ± SEM*.* Scale bars, 20 μm. *P < 0.05, between WT and R6/2 mice; ^#^*P* < 0.05 vs. water-treated R6/2 mice. **C**, **D** Striatal lysates were analyzed using Western blot analysis. The molecular mass is indicated in kilodaltons. Results were normalized to those of actin. *P < 0.05, between WT and R6/2 mice; ^#^*P* < 0.05 vs. water-treated R6/2 mice

**Fig. 6:**
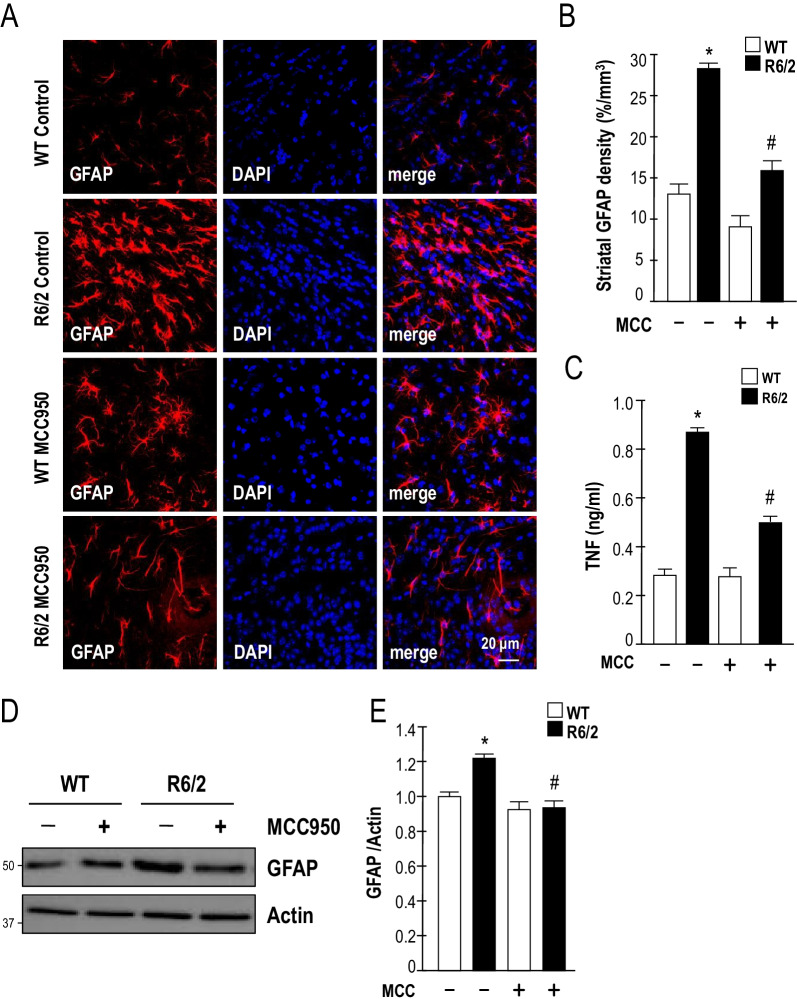
MCC950 inhibits astrocyte activation in a transgenic mouse model (R6/2) of HD. Mice were treated daily with MCC950 (10 mg/kg body weight; oral administration) or water for 5 weeks from the age of 7 weeks. **A** Brain sections of 12-week-old mice were stained against GFAP. The number of astrocytes (identified by the expression of GFAP; red) in the striatum of the indicated mice (water-treated WT mice [*n* = 6], water-treated R6/2 mice [*n* = 6], MCC950-treated WT mice [*n* = 6], and MCC950-treated R6/2 mice [*n* = 6]) were quantified. Nuclei were stained with DAPI (blue). The histograms show the integrated intensity of striatal astrocytes (**B**). At least 500 cells from each animal were counted. Data are presented as the mean ± SEM*.* Scale bars, 20 μm. *P < 0.05, between WT and R6/2 mice; ^#^*P* < 0.05 vs. water-treated R6/2 mice. **C** Striatal levels of TNF were measured using ELISA (*n* = 3–6 for each condition). Data are presented as the mean ± SEM. *P < 0.05, between WT and R6/2 mice; ^#^*P* < 0.05 vs. water-treated R6/2 mice. **D**–**E** Striatal lysates were analyzed using Western blot analysis. The molecular mass is indicated in kilodaltons. Results were normalized to those of actin. *P < 0.05, between WT and R6/2 mice; ^#^*P* < 0.05 vs. water-treated R6/2 mice

Previous studies reported that microglia are the primary mediators of neuroinflammation [40, 41]. In addition, neuroglia are key players in the pathogenesis of neurodegenerative diseases [42]. In turn, mHTT accumulation in neuronal cells has been linked to the activation of microglia [43]. R6/2 mice exhibit expression of mHTT in astrocytes, which reduced the neuroprotective effect and enhanced the TNF production of the astrocytes [44, 45]. Therefore, we examined the activation effect of long-term treatment with MCC950 on microgliosis and astrocytosis in HD. Immunofluorescence staining showed that inhibition of NLRP3 significantly reduced the number of Iba-1- (Fig. 5A, B) and GFAP-positive cells (Fig. 6A, B) in the striatum of HD mice. As shown in Figs. 5C, D, and 6D, E, Western blot analyses revealed that the elevated levels of Iba-1 and GFAP detected in R6/2 mice were reduced by MCC950. In addition, MCC950 reduced TNF production in the striatum of R6/2 mice (Fig. 6C). Collectively, these data suggest that the oral administration of MCC950 reduces neuroinflammation in a mouse model of HD. These observations support our hypothesis that the NLRP3 inflammasome plays an important role in microglial activation and astrocytosis and regulates mHTT-mediated cytokine secretion (i.e., IL-1β and TNF) in HD. The oral administration of an NLRP3 inhibitor (MCC950) increased neuronal density and reduced neuroinflammation, which were accompanied by extended lifespan and improved motor dysfunction in R6/2 mice.

## Discussion

Our results indicated that the expression of mHTT in a striatal progenitor cell and in a transgenic mouse model of HD (R6/2) enhanced the expression the NLRP3 inflammasome. To evaluate the impact of MCC950 in HD, R6/2 mice were treated daily with MCC950 (10 mg/kg of body weight; oral administration) or water for 5 weeks from the age of 7 weeks. The oral administration of MCC950 inhibits NLRP3 inflammasome assembly, reduces gliosis, and increases neuronal density in the striatum of R6/2 mice. Moreover, MCC950 improved the motor dysfunction and extended the lifespan of R6/2 mice. Our results collectively suggest that the NLRP3 inflammasome plays a critical role in the pathogenesis of HD and that MCC950 may be a potential therapeutic compound for the treatment of HD.

An excessive inflammatory reaction is one of the important factors causing neuronal death in HD. Multiple studies have shown that an exacerbated inflammatory reaction was present in patients with HD as well as mouse models of HD [25, 46, 47]. However, much of the pathological mechanisms and pathophysiological outcome of the neuronal inflammation observed in HD remain unknown. LPS was injected intraperitoneally into HD transgenic (R6/2) and WT mice, and the results showed that higher levels of TNF were produced in the plasma, liver, and brain in the LPS-stimulated R6/2 mice compared with WT mice [26]. In addition, more activated caspases were present in R6/2 mice after intraperitoneal injection of LPS compared with WT mice. IκB kinase (IKK) expression, which activates NF-κB and promotes an inflammatory reaction, was also increased in primary cultures of astrocytes from HD mice [26]. NF-κB is an essential molecule in inflammatory reactions. NLRP3 inflammasome activation requires two signals, the priming signal and the activation signal. We speculate that the mHTT-mediated activation of NF-κB may function as a priming signal, whereas the massive ATP release detected in degenerating neurons may function as an activation signal, which results in the activation of the NLRP3 inflammasome in the striatum of HD. ATP has been shown to stimulate the release and processing of IL-1β and to induce cell death in macrophages [48]. High levels of extracellular ATP lead to P2X7R-mediated glial activation and neuron–glia cross-talk. In astrocytes and microglia, large amounts of extracellular ATP induce the release of cytokines and ROS, which trigger neuroinflammation. In neuronal cells, high levels of extracellular ATP induce ion influx and neuronal cell death; moreover, additional ATP is released from neuronal presynaptic terminals into the extracellular space [49]. Extracellular ATP has recently been shown to cause neuronal cell death through stimulation of P2X7 receptors. SH-SY5Y cells treated with 6-OHDA (30 μM) triggered a rapid and sustained increase in extracellular levels of ATP. Rats treated with 6-OHDA in the striatum for 19 days showed an increase in the evoked release of ATP [50]. P2X7R stimulation is known to cause K^+^ efflux, which may trigger NLRP3 inflammasome activation and secretion of inflammatory cytokines (e.g., IL-1β, IL-18, and TNF-α) from astrocytes and microglia. Moreover, stimulation of P2X7R increases Ca^2+^ influx and leads to ATP and glutamate release from nerve terminals and astrocytes, which is responsible for excitotoxicity [49]. Increased protein levels of the P2X7 receptor in the striatum have been reported in two different HD mouse models, namely R6/2 and Tet/HD94 [51], and in patients with HD [52]. P2X7R is considered a potential target for therapeutic intervention in HD; indeed, in vivo administration of a P2X7R antagonist (Brilliant Blue-G) has been shown to prevent neuronal cell death and attenuate body-weight loss and motor-coordination deficits [51]. However, ATP release in HD is yet to be explored. To address this, further research in this area is required.

Three neurodegenerative diseases are characterized by the abnormal aggregation of proteins in the central nervous system (CNS), i.e., Aβ, α-synuclein, and mHTT, which cause the pathogenesis of IL-1β in and progression of AD, PD, and HD, respectively. In HD and other neurodegenerative diseases caused by aberrant aggregation of proteins including AD and PD, the NLRP3 inflammasome participates in disease progression. In AD, Aβ induces NLRP3 inflammasome activation in primary microglia, which is required for the Aβ-induced activation of caspase-1 and release of IL-1β [17, 53]. Moreover, α-synuclein aggregates have been suggested to be a potential activator of the NLRP3 inflammasome [54]. NLRP3 and caspase-1 are significantly enhanced in 13-week-old R6/2 mice and mediate pyroptotic cell death in HD [55]. This is consistent with our finding that mHTT is involved in the activation of the NLRP3 inflammasome in HD (Figs. 1E, 2A, E). Inhibition of the NLRP3 inflammasome using an NLRP3 inhibitor reduced the expression of ASC, cleavage of caspase-1, and production of IL-1β in R6/2 mice (Fig. 2C–J). The activation of caspase-1 was shown both in vitro [56] and in vivo [57], potentially contributing to the neurodegeneration observed in HD. Ona et al. found that caspase-1 inhibition in R6/2 mice can alleviate the disease course [57]. Inhibition of microglial galectin-3 expression can effectively decrease inflammatory responses in cells, alleviate the symptoms of neurodegenerative diseases in animal models, and increase the lifespan of R6/2 mice. Previous studies found a high level of galectin-3 and damaged lysosomes in the microglia of HD mice [25], which induces an increase in downstream NLRP3 expression and results in the release of IL-1β, thereby worsening neuroinflammation. Galectin-3 inhibition can effectively improve lysosome clearance in cells and decrease the inflammatory reaction in microglial cells [25]. These results suggest that the NLRP3 inflammasome is one of the factors that worsen disease progression in neurodegenerative diseases.

MCC950 oral dosing in mice (ED50 of ~ 15 mg/kg) attenuated IL-1β secretion in vivo [58]. The in vivo concentrations of IL-1β following MCC950 administration in mice were attenuated by 50% with 0.4 mg/kg, 90% with 1.2 mg/kg, and > 90% with > 4 mg/kg, thereby establishing the capability of this molecule [59]. Although MCC950 is not currently used in the treatment of HD, it shows a certain level of protective effect in other neurological diseases. We searched the literature for the usage of MCC950 in mice. We found that MCC950 has been used in several CNS diseases, e.g., 10 mg/kg of MCC950 in AD (for 3 months) [60], 10 mg/kg in aged mice (for 2 days) [61], 10 mg/kg in EAE (for 9 days) [62], 20 mg/kg in PD (for 21 days) [63], and 50 mg/kg after traumatic brain injury (at 1 and 3 h post-TBI) [64]. Intramuscular injection of MCC950 (10 mg/kg) to APP/PS1 mice can effectively decrease the production of TNF and IL-1β while simultaneously decreasing Aβ accumulation in the brain with improved cognitive functions in mice [60]. Oral administration of MCC950 (20 mg/kg) protected the dopaminergic cells in the brains of PD mice and improved their behavioral deficits [63]. In our study, we administered MCC950 daily for 5 weeks. Since this is not a short-term dosing, we used 10 mg/kg MCC950 in R6/2 mice. We tested two batches of R6/2 mice in our experiments and the results were consistent, i.e., systematic administration of 10 mg/kg of MCC950 to R6/2 mice suppressed the NLRP3 inflammasome, reduced neuroinflammation, extended the lifespan, and improved motor dysfunction. MCC950 reduces IL-1β production in vivo and attenuates the severity of a multiple sclerosis mouse model (experimental autoimmune encephalomyelitis mice). Furthermore, treatment with MCC950 rescues neonatal lethality in a genetic mouse model of cryopyrin-associated periodic syndrome [21]. Traumatic brain injury (TBI) upregulates NLRP3, ASC, cleaved caspase-1, and IL-1β in the perilesional area. MCC950 (50 mg/kg, intraperitoneally) treatment resulted in a significant improvement in neurological function and reduced cerebral edema in animals with TBI. MCC950 was shown to block caspase-1 cleavage and IL-1β production in TBI. MCC950 treatment also reduces lesion volume and improves motor and cognitive functions after TBI [64, 65]. MCC950 preserves the blood–brain barrier (BBB) and decreases cell death by downregulating NLRP3, caspase-1, and IL-1β production in mice with intracerebral hemorrhage. MCC950 has therapeutic potential in in mouse model of transient middle cerebral artery occlusion (tMCAO) via the decrease of TNF, caspase-3 cleavage, and phosphorylated NFκB–p65 and IκBα levels[66]. Our results also indicated that MCC950 downregulated the phosphorylation level of IκB and P65 in BV2 cells and in the striatum of R6/2 mice (Additional file 1: Fig. S3).

**Fig. 7:**
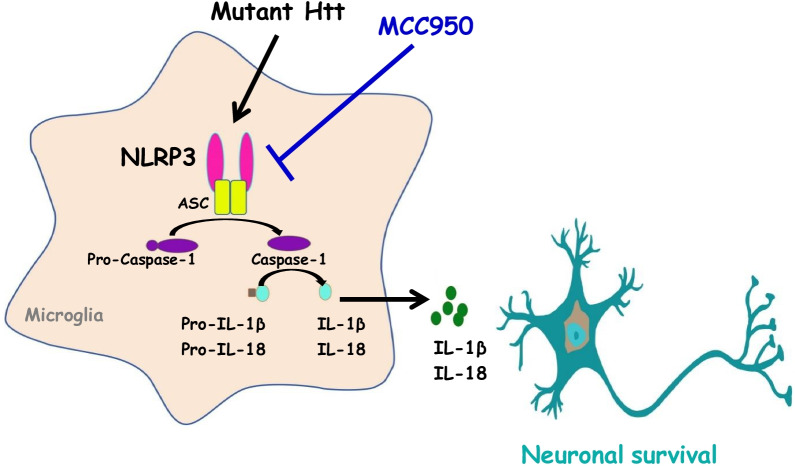
Schematic representation of signaling pathways that mediate the action of MCC950 in rescuing the detrimental effect of the NLRP3-dependent pathway in the presence of mutant HTT. In the current study, we demonstrated that expression of polyQ-expanded mHTT enhanced the activation of the NLRP3 inflammasome in striatal progenitor cell lines (STHdh^Q7^and STHdh^Q109^) and a transgenic mouse model of HD (R6/2 mice). Long-term treatment of HD mice with a NLRP3 inhibitor (MCC950) not only reduced the activation of the NLRP3 inflammasome but also rescued neuronal survival and reduced gliosis in a transgenic mouse model (R6/2) of HD. Importantly, oral administration of MCC950 markedly reduced disease progression in R6/2 mice. Collectively, these findings indicate that MCC950 has therapeutic potential for the treatment of HD. Further characterization of the functional role of MCC950 in HD, as proposed herein, will provide important insights that could help facilitate the development of neuroprotective strategies targeting the bioenergetic defects of HD and other aggregate-related diseases

Microglia and astrocytes play an important role as the immune effectors in the CNS. Our data showed that inhibition of NLRP3 by MCC950 reduced microgliosis and astrocytosis in the striatum of R6/2 mice, leading to downregulation of IL-1β and TNF (Figs. 2C, D, and 6C). Fu et al. found that the administration of MCC950 reduced the surgery-induced increase in GFAP- and Iba1-positive cells [61]. In addition, MCC950 administration also reduced the necrotizing-enterocolitis-enhanced Iba-1- and GFAP-positive cells in both the hippocampus and cerebral cortex [67]. Taken together, these previous results suggest that, as well as microgliosis, inhibition of NLRP3 also reduces astrocytosis in the CNS, consistent with our observations that MCC950 not only inhibited microglia activation but also reduced astrocytosis in HD (Figs. 5, 6). The results presented in this study support the hypothesis that the prevention of NLRP3 inflammasome activation by MCC950 is beneficial to striatal cells in a transgenic mouse model of HD (Fig. 7).

## Conclusions

In conclusion, we demonstrated that expression of polyglutamine-expanded mHTT enhanced the activation of the NLRP3 inflammasome in the striatum of HD mice. The NLRP3 inflammasome caused the activation of caspase-1 and the release of IL-1β, resulting in inflammation and leading to neuronal cell death in HD. Moreover, we further demonstrated that long-term treatment of HD mice with an NLRP3 inhibitor (MCC950) not only reduced the activation of NLRP3 and ROS production, but also rescued neuronal survival and attenuated gliosis in HD. Oral administration of MCC950 halted the disease progression and markedly enhanced lifespan in a transgenic mouse model (R6/2) of HD through the inhibition of the NLRP3 inflammasome overactivation pathway. Collectively, our findings suggest that MCC950 is a potential therapeutic treatment for HD.

## Supplementary Information

**Additional file 1: Figure S1.** MCC950 markedly reduces cytotoxicity in striatal progenitor cells. **Figure S2.** MCC950 markedly reduced IL-18 secretionin BV2 microglial cells and in a transgenic mouse model (R6/2) of HD. **Figure S3.** MCC950 downregulated the phosphorylation level of Iκ-B and P65.

## Data Availability

All data of this study are included in the manuscript.

## Abbreviations

3-NP: 3-Nitropropionic acid
AD: Alzheimer’s disease
CNS: Central nervous system
GFAP: Glial fibrillary acidic protein
HD: Huntington’s disease
*HTT*: Huntingtin
mHTT: Mutant HTT
Iba-1: Ionized calcium-binding adaptor molecule-1
IKK: IkB kinase
IL-1β: Interleukin‐1β
LKB1: Liver kinase B1
LPS: Lipopolysaccharide
NLRP3: NACHT, LRR, and PYD domains-containing protein 3
PD: Parkinson’s disease
TBI: Post-traumatic brain injury
WT: Wild-type
