# Supplementary material for: A selective inhibitor of the NLRP3 inflammasome as a potential therapeutic approach for neuroprotection in a transgenic mouse model of Huntington’s disease
Source: J Neuroinflammation. 2022 Feb 26;19:56. doi: 10.1186/s12974-022-02419-9 (PMC8882273; doi:10.1186/s12974-022-02419-9)
Supplement: Supplementary file 1 — Additional file 1: Figure S1. MCC950 markedly reduces cytotoxicity in striatal progenitor cells. Figure S2. MCC950 markedly reduced IL-18 secretionin BV2 microglial cells and in a transgenic mouse model (R6/2) of HD. Figure S3. MCC950 downregulated the phosphorylation level of Iκ-B and P65. [file 12974_2022_2419_MOESM1_ESM.pdf]

Additional Data

A selective inhibitor of the NLRP3 inflammasome as a potential therapeutic approach for neuroprotection in a transgenic mouse model of Huntington's disease

Kai-Po Chen[1#], Kuo-Feng Hua[2,3#], Fu-Ting Tsai[1], Ting-Yu Lin[1], Chih-Yuan Cheng[1], Ding-I Yang[4], Hsien-Ta Hsu[5,6], and Tz-Chuen Ju[1,7*]

[#]These authors contributed equally.

*** Corresponding author: Tz-Chuen Ju**, E-mail: tzchuen@thu.edu.tw.

Inventory of Additional Data

Additional Figures and Legends

- Additional Figure 1. MCC950 markedly reduces cytotoxicity in striatal progenitor cells.
- Additional Figure 2. MCC950 markedly reduced IL-18 secretion in BV2 microglial cells and in a transgenic mouse model (R6/2) of HD.
- Additional Figure 3. MCC950 downregulated the phosphorylation level of Iκ-B and P65.

Additional Figures and Legends
Figure 1

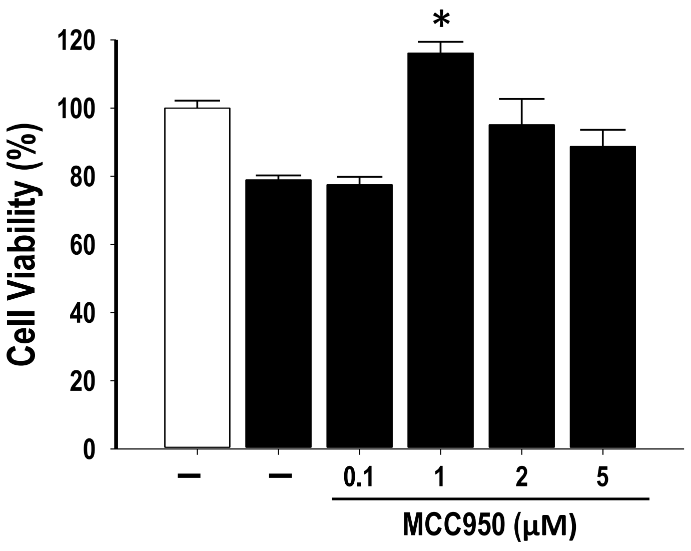

Fig. 1. MCC950 markedly reduces cytotoxicity in striatal progenitor cells. ST*Hdh*^{Q109} cells were incubated for 24 h with MCC950, cell death was quantified using the CCK-8 assay. The data are presented as the mean ± SEM from three independent experiments. *$P < 0.05$ vs. untreated ST*Hdh*^{Q109} cells.

Additional Figures and Legends
Figure 2

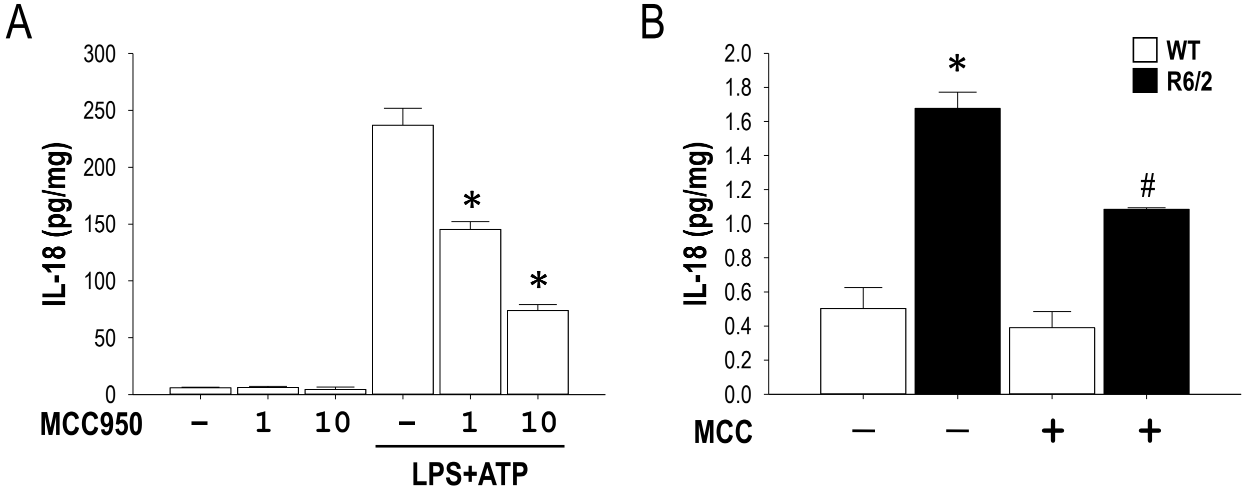

Fig 2. MCC950 markedly reduced IL-18 secretion in BV2 microglial cells and in a transgenic mouse model (R6/2) of HD. (A) BV2 microglia were incubated for 2 h with LPS (1 μg/mL) followed by incubation with MCC950 (1 and 10 μM) for 2 h. The cells were then incubated with ATP (1 mM for 6 h). IL-18 expression levels were measured using the ELISA. **P* < 0.05 compared to LPS and ATP treated cells (n = 3 for each condition). (B) Mice were treated daily with MCC950 (10 mg/kg of body weight; oral administration) or water for 5 weeks from the age of 7 weeks. The levels of IL-18 in the striatum were measured by ELISA (*n* = 3 for each condition). The data are presented as the mean ± SEM. **P* < 0.05, between WT and R6/2 mice; #*P* < 0.05 vs. water-treated R6/2 mice.

Additional Figures and Legends
Figure 3

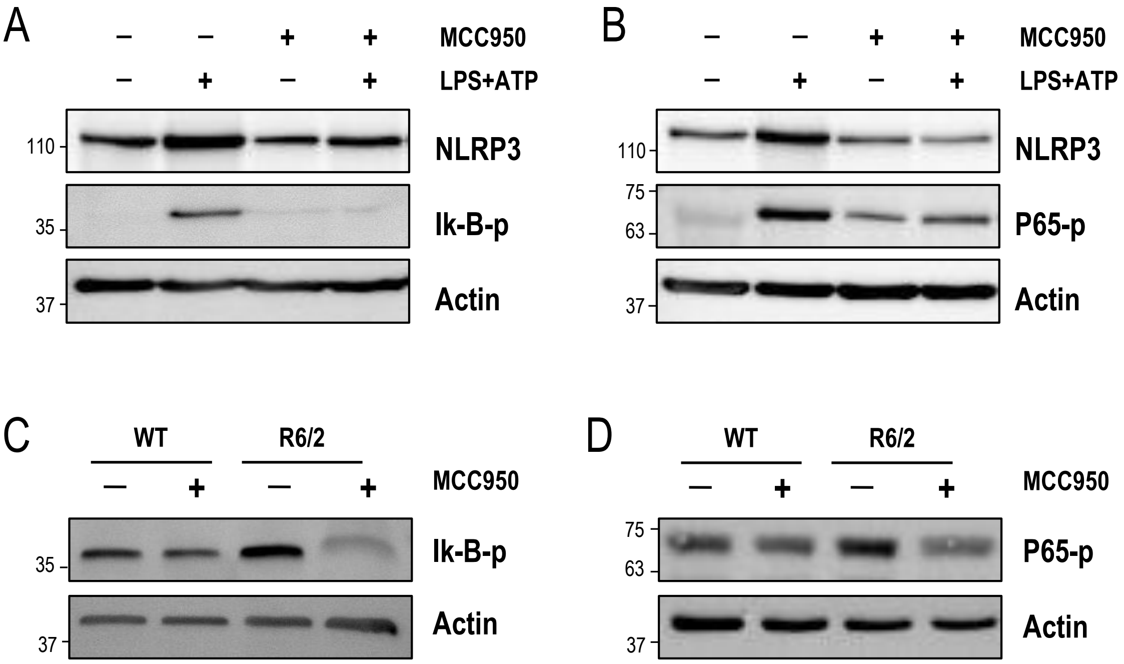

Fig 3. MCC950 downregulated the phosphorylation level of Iκ-B and P65. (A, B) BV2 microglia were incubated for 4 h with LPS (1 µg/mL) followed by incubation with MCC950 (1 µM) for 2 h. The cells were then incubated with ATP (1 mM for 24 h). Total lysates of BV2 cells were assessed by western blot analysis to determine the phosphorylation levels of Ik-B and P65. The molecular mass is indicated in kilodaltons. (C, D) Mice were treated daily with MCC950 (10 mg/kg of body weight; oral administration) or water for 5 weeks from the age of 7 weeks. Striatal lysates were analyzed using western blot analysis. The molecular mass is indicated in kilodaltons.
